# Narrowing the coordination solution space during motor learning standardizes individual patterns of search strategy but diversifies learning rates

**DOI:** 10.1038/s41598-023-29238-z

**Published:** 2023-02-03

**Authors:** John Komar, Ludovic Seifert, Nicolas Vergne, Karl M. Newell

**Affiliations:** 1 Physical Education and Sports Science, National Institute of Education, Nanyang Technological University, 1 Nanyang Walk, Singapore, 637616 Singapore; 2 Physical Education and Sports Science, CETAPS, UNIROUEN, Normandie University, Mont Saint Aignan Cedex, France; 3 Laboratoire de Mathématiques Raphaël Salem, UMR CNRS 6085, Université de Rouen, Normandie University, Mont-Saint-Aignan, France; 4 Department of Kinesiology, University of Georgia, Athens, GA USA

**Keywords:** Human behaviour, Motor control

## Abstract

Constraints on practice can benefit motor learning by guiding the learner towards efficient coordination patterns, but can also narrow the potential solution space of coordination and control. The aim of this paper was to investigate whether narrowing the solution space through more restrictive task constraints limits the expression of potential exploratory behaviours during the learning process, identified using Drifting Markov Models. In a breaststroke swimming task, the change in interlimb coordination of 7 learners practicing for 16 lessons over 2 months was analysed to quantify motor exploration and identify periods of metastable regimes of coordination. Results showed that the observed exploratory dynamics were highly individual both in terms of range of exploration and in the patterns of search. The more restrictive task constraints did not impair the amount of exploration but rather channelled the exploration around a few selected patterns. In addition, restraining the nature of the exploratory process increased the inter-individual differences of the learning rate. Although manipulating the task constraints during learning can help learners to escape from the behavioural consequences of their intrinsic dynamics, maintaining a broad solution space for a diversity of coordination patterns to emerge was key to fostering effective exploration of individual coordination solutions.

## Introduction

The study of motor learning as a process of active exploration of a workspace was initially proposed by Gel’fand and Tsetlin^1^ and elaborated into a perception-action framework for exploration in movement coordination and control^2–4^, a perspective that has gained renewed attention^5–7^. Exploration reflects the navigation through the perceptual-motor workspace in a search for an effective and efficient task solution^2^. With practice and learning, the perceptual-motor workspace evolves qualitatively as the temporary stabilization of newly explored coordination patterns that allows for subsequent exploration^8,9^. Indeed, the ecological approach to perception and action exploration is defined as a continuous and active process through which the learner differentiates and picks up information in the control of action^10^. In other words, exploration is a process of information gathering from the search for solutions within the perceptual-motor workspace^5^.

Exploration is a search through the space of possibilities to adapt to the most useful information in achieving the task goal. This exploration has been associated with variability in the movement or the outcome of the task^6^. In redundant systems, the learner has the opportunity to explore a larger set of different motor solutions in order to reach the task goal^8^. In this regard, exploration between those motor solutions has been viewed on a continuum, i.e., as a balance between variability and stability rather than merely an increased variability between two different behaviors^7^.

This perspective is in line with the concepts of exploration and exploitation from reinforcement learning theory, where an optimal ratio between exploration and exploitation (or stabilization) during practice has been shown to lead to better performance^11–13^. Trial-to-trial motor variability is typically seen as a reflection of exploration that is essential for motor learning^14^ and dynamically regulated based on the outcome of previous trials to promote motor learning^15,16^. Uehara et al.^17^ found that exploration could remain elevated to facilitate learning in subsequent training blocks. More specifically looking at the acquisition of coordination, for example in a football kicking task, Chow et al.^18^ showed how a learner can search for functional coordination patterns trial after trial, with both periods of relative stability and periods of high variability.

Using a similar methodology, Komar et al.^19^ quantified the ratio between trial-to-trial variability (i.e., considered as exploration of the perceptual motor workspace) and trial-to-trial stability (i.e., considered as exploitation of existing coordination patterns). This ratio represents the balance between exploration and exploitation of coordination pattern during learning, although an *optimal* ratio was not presented. However, the absence of considering the qualitative nature of exploration (i.e., which behaviors are explored and how) remains a limitation, since that exploration was quantified as a switch between different coordination patterns without looking at the nature of the transitions.

Gel’fand and Tsetlin^1^ described three types of exploration strategy in system control (see also^2^). One is a blind strategy, where all points of the perceptual-motor workspace are explored in a random order until a functional solution is found, i.e., the direction of the exploration is not based on the result of the previous exploration. Another strategy was named local and corresponds to a continuous and directed search towards the functional solution; moreover, the exploration at time *t* is dependent on the result of the previous exploration at time *t − *1 as the learner gets linearly closer and closer to the functional solution proportionally with practice quantity. The past has a certain effect on the exploration in this strategy that leads to the progressive discovery of expert behavior. The third strategy was non-local or hybrid and corresponds to a discontinuous search. This means that a temporary anchoring point is made within the workspace and exploration then continues around this point until another anchor emerges and is explored. From this perspective, individual learners can exhibit different strategies and each learner can also change her/his own strategy through the learning process. However, a ratio quantifying the global percentage of switches between behaviors during learning is not relevant to identifying learning strategies. Rather, investigating the process of learning necessitates examining the convergence/divergence towards/from specific behaviors and how those evolve with practice (i.e., how some specific patterns are progressively anchored and how some are forgotten). In other words, it would be necessary to deeply look at the transition over time between patterns and how those transitions are organized and evolve, for which Markov Modelling could help.

If the deployment of augmented constraints on practice can benefit learning by effectively guiding exploration^20^, those constraints can also limit the expression of potential innovative movement solutions as they tend to restrict the diversity of emerging movement solutions^21^. Therefore, there is an intimate relation between the range of exploration (i.e., how many different patterns can be explored) and the qualitative nature of exploration (i.e., how the exploration happens) during practice and learning. The aim of this paper was to identify and quantify the exploratory strategies during learning in a task where the original level of constraint is relatively low such that the exploratory behaviour can be observed. A secondary aim was to investigate whether the manipulation of more restrictive task constraints to the coordination solutions impacts the expression of potential exploratory behaviours and strategies during learning. Lastly, the relationship between motor exploration and performance improvement was investigated.

Breaststroke swimming requires the development of a multi-articular coordination where a variety of forms of coordination can theoretically emerge, although a biomechanically optimal pattern of coordination between elbow and knee oscillations is adopted by expert performers compared to novices^22^. For example, experts were able to start a swimming cycle with arm–leg coordination in anti-phase followed by an in-phase mode and then back to anti-phase mode through every cycle of movement (i.e., every 1–2 s). In aquatic activities where the environmental constraints play an important role (e.g., due to high density of water), the use of an optimal coordination pattern becomes pertinent for efficient performances^23,24^. Although the coordination pattern is a key factor in swimming performance (i.e., whether the performance is about swimming faster or swimming more efficiently), this coordination pattern has to match multiple factors to reach high performance, e.g., swimmers’ energetic characteristics^25–27^, anthropometric variables, or even swimming specialty (e.g., long versus short distance swimmers)^28^. Therefore, the acquisition of an optimal coordination pattern is only one of the multiple interacting factors for performance improvement in swimming^26^. Interestingly in swimming, the level of environmental constraint (i.e., the amount of forward resistance swimmers have to overcome in order to move) is related to the swimming speed squared^29^, therefore an increase in swimming speed leads to quadratic increase of water resistance as an environmental constraint.

The first hypothesis examined was that an exploratory process would reflect a balance between flexibility and stability of the movement dynamics and focusing on the transitions between patterns (i.e., using Markov chains) allows the identification of different search strategies. Our second hypothesis was that more restrictive task constraints would impact the opportunities for exploration, both in lowering the quantity of exploration and in the nature of the exploration (i.e., how much is explored and what is explored and how). In that sense, a limited number of exhibited patterns by a participant (i.e. a limited diversity in the patterns) would be associated with a lower level of exploration. The final hypothesis examined was that a limited quantity of exploration during the learning process would slow the rate of improvement in performance over the practice period.

## Results

During the entire learning process (i.e., during each session and trial/repetition), we recorded the learner inter-limb coordination and associated outcome in terms of performance (i.e., distance covered per cycle). Using a clustering of movement coordination and a modelling of learning dynamics through Drifting Markov Models, we quantified the level of motor exploration happening during learning as well as the dynamics of the performance improvement.

### Performance outcome

**Figure 1:**
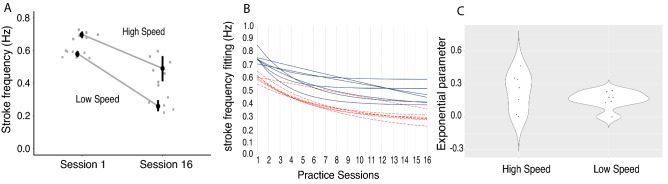
Performance improvement. (**A**) Stroke frequency during session 1 (pre-test) and session 16 (post-test) for both speed condition; (**B**) Exponential model presenting the individual rate of learning in the high-speed condition (black continuous line) and low-speed condition (red dashed line). (**C**) Exponential parameter values showing the interindividual variability in the rate of learning between high speed and low speed conditions.

The two-way ANOVA showed a significant main effect of session with a large effect size, F(1, 6) = 243.790, *p* < 0.001, η_p_^2^ = 0.976, achieved power = 0.99, as well as a main effect of speed condition with a large effect size, F(1, 6) = 74.843, *p* < 0.001, η_p_^2^ = 0.926, achieved power = 0.99. Interestingly, although marginal, a significant interaction effect appeared with *p* = 0.050 (F(1, 6) = 6.001, *p* = 0.050, η_p_^2^ = 0.500, achieved power = 0.99). The swimmers, therefore, showed a higher stroke frequency when swimming at higher speeds during the first lesson (Mean difference (MD) between low and high speed in session 1 = − 0.134 Hz, 95% Confidence Interval (CI) = [− 0.222; − 0.045]) as well as the last lesson (MD between low and high speed in session 16 = − 0.226 Hz, 95% CI = [− 0.314; − 0.137]). However, the interaction reveals that the decrease in stroke frequency was more prevalent in the low-speed condition (MD between session 1 and session 6 in low speed = 0.316 Hz, 95% CI = [0.236; 0.397]) compared to high-speed condition (MD between session 1 and session 6 in high speed = 0.224 Hz, 95% CI = [0.144; 0.305]) (Fig. 1.A).

Figure 1.B and Table 1 show the learning rate from each individual exponential fitting as well as the r^2^ and RMSE values showing the quality of the fitting. After checking for normally distributed values, the paired-sample *t*-test performed on the learning rate did not reveal a significant difference between the two speed conditions (*t*(6) = − 1.373, *p* = 0.219) (Fig. 1.B). However, the dispersion of the values within the speed conditions (Fig. 1.C) showed a more similar learning rate between the participants in the low-speed condition compared to the high-speed condition. Indeed, a Levene’s test for equality of variance on the exponential exponent values showed an unequal variance between both speeds (F(1, 12) = 6.070, *p* = 0.030), with the variance within the low speed condition (VARlow = 0.00644 ± 0.07930) being smaller than the variance within the high speed condition (VARhigh = 0.03042 ± 0.02031).

**Table 1:** Parameters of the individual exponential models and indicators of quality of the fitting for both high speed and low speed conditions.

	Participants						
	1	2	4	5	6	7	8
*Low speed*							
b value	0.0006178	0.1777	0.1394	0.1796	0.2285	0.1286	0.2407
r^2^	0.9374	0.8805	0.8935	0.897	0.8342	0.9576	0.95
rmse	0.0233	0.0404	0.0317	0.0427	0.045	0.0261	0.0247
*High speed*							
b value	0.02319	0.3423	0.005785	0.2656	0.1559	0.3524	0.4648
r^2^	0.7468	0.8571	0.8777	0.9159	0.7573	0.693	0.6866
rmse	0.0484	0.0467	0.0402	0.0442	0.0642	0.0338	0.0484

### Coordination profiling

**Figure 2:**
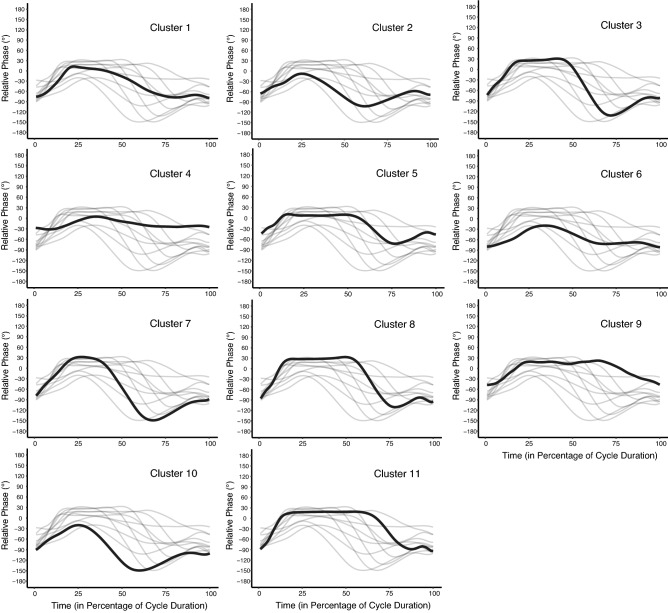
Coordination profiles. Average coordination patterns for each of the 11 emerging clusters (the grey backgrounds represent the 10 other profiles as reference).

The output of the cluster analysis showed the emergence of 11 different arm-leg coordination patterns throughout the learning phase (i.e., during the 2 months) (Fig. 2). The individual dynamics of those patterns cycle per cycle are presented in supplemental material. The BIC criterion ([2–16] potential clusters) showed that the optimal number of clusters that best fit the data was 11. Indeed, 11 clusters corresponded to the first value of the plateau* in the BIC vector [BIC = − 12266770; − 12054679; − 11835712; − 11758107; − 11478308; − 11414261; − 11299105; − 11096673; − 10771125; − 10382736*; − 10477354; − 10527102; − 10513261; − 10358955; − 10398427].

### Quantity of exploration

**Figure 3:**
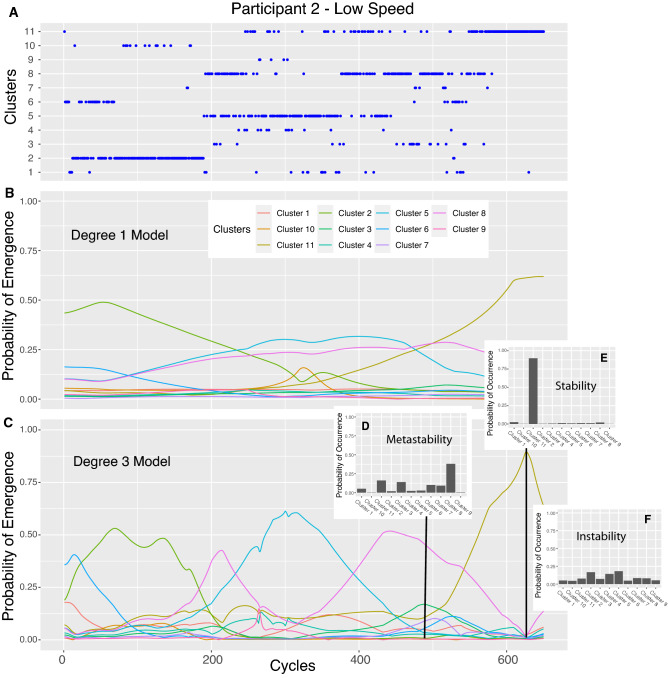
Individual dynamics of learning and exploration for Participant 2. (**A**) Cycle per cycle dynamics (1 dot representing one performed cycle) of coordination patterns during the entire process of learning (from cycle 1 to cycle 680). (**B**) Simulation of Drifting Markov Modelling trained on data in (**A**) showing what is the probability of appearance of each pattern during learning with a linear modelling (i.e., degree 1) of the probabilities of transition between patterns. (**C**). Simulation of Drifting Markov Modelling trained on data in (**A**) showing what is the probability of appearance of each pattern during learning with a polynomial modelling of degree 3 of the probabilities of transition between patterns. For each pattern, the quantity of exploration is computed as the distance between those two models in (**B**) and (**C**). (**D**) A section of the learning dynamics (cycle 488) showing a metastable regime with few patterns that are not really stable neither really unstable. (**E**) A section of the learning dynamics (cycle 644) showing a period of stability with one highly probable pattern. (**F**) A section of the learning process (from participant 6) showing instability with an evenly distributed probability of appearance of patterns.

**Figure 4:**
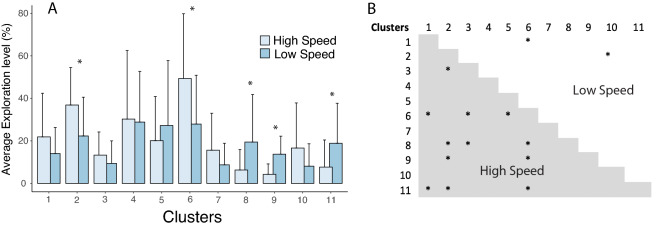
Effect of speed and pattern on exploration. (**A**) Effect of speed condition on the exploration quantity of each specific coordination pattern, the stars showing the patterns that are explored with significant different quantities between both conditions (at *p* < .05). (**B**) Effect of pattern within each speed condition, the starts showing which pairs of patterns are explored with a significantly different quantity within a specific speed condition (at *p* < .05).

The DMM outputs are presented in Fig. 3A, B and C for the participant 2 in the low speed condition, the modelling for all the participants and speed conditions is included in Online Appendix B. Regarding the quantity of exploration per cluster and for the two speed conditions, the two-way ANOVA showed no significant effect of speed between the two conditions, but a significant main effect of the clusters (F(10, 60) = 2.537, *p* = 0.013, η_p_^2^ = 0.297, achieved power = 0.99) and also a significant interaction between cluster and speed condition with a large effect size, F(10, 60) = 2.678, *p* = 0.009, η_p_^2^ = 0.309, achieved power = 0.99. More precisely, post-hoc Bonferroni tests showed that the quantity of exploration was significantly different between the two speed conditions only for cluster 2 (MD between low and high speed for cluster 2 = − 17.41%, 95% CI = [− 25.44; − 0.9.38]), cluster 6 (MD between low and high speed for cluster 6 = − 26.54%, 95% CI = [− 51.67; − 1.42]), cluster 8 (MD between low and high speed for cluster 8 = 13.73%, 95% CI = [0.76; 26.69]), cluster 9 (MD between low and high speed for cluster 9 = 9.46%, 95% CI = [4.56; 14.36]) and cluster 11 (MD between low and high speed for cluster 11 = 12.33%, 95% CI = [1.97; 22.71]) (Fig. 4A) (all *p*_s_ < 0.045). All the other clusters were not differentially explored by both speeds.

**Figure 5:**
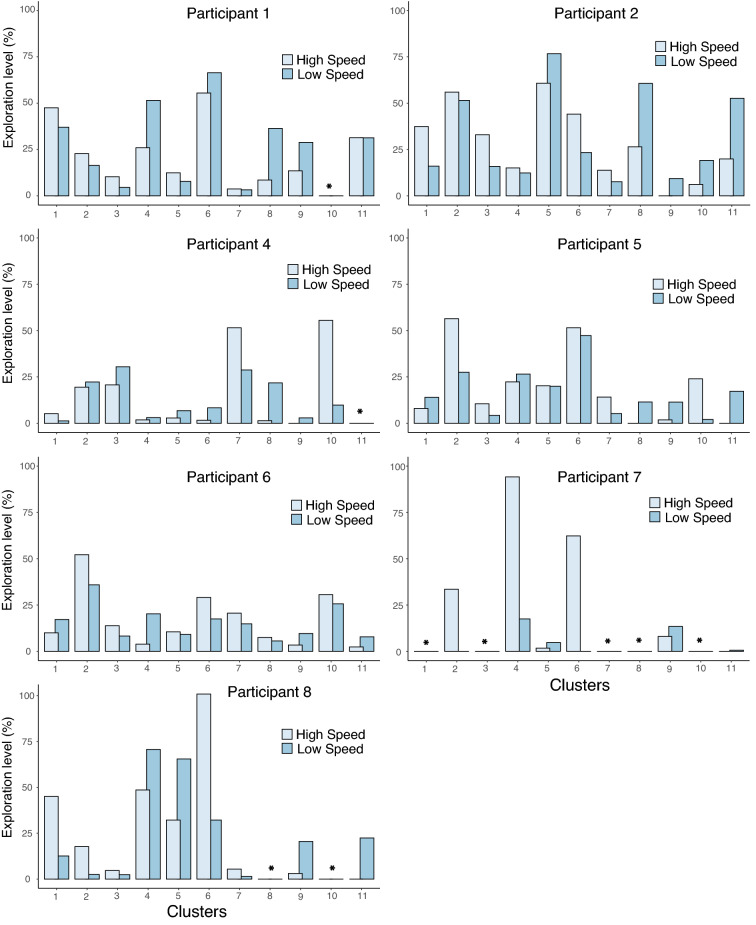
Exploration quantity. Individual quantities of exploration for each participant and each pattern in both low and high-speed conditions (*indicates a pattern that was never visited by this learner).

On the other hand, Bonferroni post-hoc tests showed that the usage of the different clusters within each speed condition was not similar with a more equal usage of the clusters in the low-speed condition. Indeed, in high-speed condition, 12 significant differences can be identified between clusters’ quantity of exploration, whereas only 2 significant differences appeared in the clusters’ quantity of exploration in the low-speed condition (Fig. 4B). More precisely cluster 6 and cluster 2 were explored more than others in the high-speed condition whereas clusters 8, 9 and 10 appeared less explored. Conversely, in the low-speed condition, only two significant differences between the quantity of exploration of clusters appeared (Figs. 4 and 5 for the individual distribution of exploration per cluster). The within-participant variability in exploration quantity between the clusters (i.e., standard deviation) is lower for low speed condition (mean SD = 15.61% ± 7.79) compared to higher speed condition (mean SD = 22.36% ± 6.69) (*t*(6) = − 2.591, *p* = 0.041, d = − 0.979), revealing a more equally distributed amount of exploration across the different clusters at low speeds and conversely a more selective exploration at faster speeds.

Looking at individual dynamics of exploration (see online appendix B for the full dynamics of every participant), participants with low level of exploration exhibited two distinct dynamics. On one hand some participants exhibited a very strong pattern that remained all over the practice time (i.e., very high probably of appearance during the 16 sessions) (e.g., participant 7 in slow speed condition). On the other hand, some participants exhibited a lack of (even temporary) stability, showing random-like fluctuations of their behaviour all over the 16 sessions (e.g., participant 6 in slow speed condition). Indeed, those two dynamics made the degree 1 and degree3 DMM very similar, therefore leading to a very low level of exploration.

### Relationship between number of emerging patterns, quantity of exploration, performance improvement and learning rate

**Figure 6:**
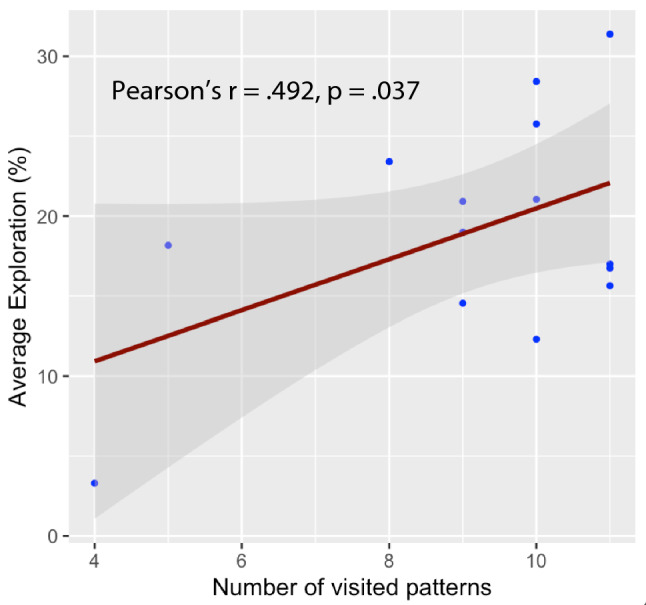
Relationship between emerging patterns and exploration. Significant correlation between the number of different patterns visited and the amount of exploration, the higher the number of different patterns the higher the amount of exploration.

Following our hypothesis, participants who had lower number of different coordination patterns visited were more likely to show a low range of exploration (Pearson’s *r* = 0.492, *p* = 0.037, Fig. 6). However, exhibiting a large number of different coordination patterns during learning was not correlated with a greater improvement in performance between first and last session (Pearson’s *r* = 0.256, *p* = 0.811), neither a higher quantity of exploration during learning was correlated with a higher improvement in performance between first and last session (Spearman’s rho = − 0.052, *p* = 0.43). Similarly, no significant correlation appeared between the number of different visited patterns and learning rate (Spearman’s rho = − 0.212, *p* = 0.77) nor between the quantity of exploration and the learning rate (Pearson’s *r* = 0.234, *p* = 0.21).

## Discussion

The aim of this paper was to quantify and identify different exploratory strategies during motor learning and investigate whether a more restrictive set of task constraints can limit the expression of potential exploratory behaviours during the process of motor learning.

On the basis of Drifting Markov Modeling on the individual learning dynamics, we propose a definition of exploratory activity in learning based on the nonlinear evolution of the probabilities of transiting towards specific coordination patterns, with successive increase and decrease of those probabilities of transition. In this regard, the degree three DMM highlighted those appearance and disappearance of coordination patterns in the dynamics of learning (Fig. 3). At some points during the learning process, coordination patterns strongly appeared through high probability of transiting towards those specific patterns, whereas later on those patterns totally disappeared for other coordination patterns to appear. In this regard, the process of learning involved the emergence of three different but intertwined dynamics: i) coordination patterns that disappeared early in practice (i.e. initial behaviors that were abandoned early during practice), ii) coordination patterns that appeared with practice (i.e. a coordination pattern that appeared with practice and that was stabilized in the latest sessions), iii) coordination patterns that were explored (i.e. coordination patterns that showed high or even the highest probability of transition at some point of the learning process, but that totally disappeared later on).

The coordination patterns that successively appeared and disappeared represent an *exploratory dynamics*, reflected by a temporary visit of a specific coordination patterns in order to gather functional information^8^. Those explored coordination patterns are strongly anchored for a period of time (i.e., with the highest probability to transit towards them), but still are not the final destination of the learner, which fits the definition of a local strategy^1^. This exploration dynamics, as an information gathering process is more than merely a global trial to trial variability of the behavior during practice as proposed previously^19,30^. Indeed, a global ratio between the transition of coordination patterns between trials and the repetition of similar coordination patterns between trial (e.g., the exploration/exploitation ratio^19,30^) does not account for this exploratory dynamics during learning as it does not account for the order or sequencing of the transitions.

In the present study, we propose that the quantity of exploration that occurs during learning resides in the evolution of the probabilities of transiting from any previous pattern towards another specific one. In other words, during learning, the learner could on one hand show, trial after trial or session after session, a linear increase in the probability of transiting towards the to-be-learned pattern expressed through a linear DMM modeling. On the other hand, the learner could show fluctuations in the probabilities of appearance of a specific behavior during the process of learning. Eventually, the Euclidean distance between a degree one DMM modeling (i.e., a linear evolution of the behavior) and the degree three DMM modeling (i.e., representing the potential three different dynamics) represents the quantity of exploration that occurred during learning. In that sense, the level of exploration of a coordination pattern is, therefore, not anymore represented by the recurrence of this pattern during learning, but by the fluctuations of the probabilities of transition towards this specific pattern in reference to what those probabilities are in a linear learning model. Motor exploration is, therefore, quantified as the amount of divergence of the behavior from a linear appearance/disappearance of coordination patterns.

Looking at the individual level of exploration, the individual nature of the exploratory process appears. Participants who showed a high amount of exploration are the participants who exhibited a temporary stability of one specific coordination pattern followed by its disappearance (e.g., participants 1, 2, 5, 8). On the other side, participants who showed a low level of exploration are the ones who exhibited either *i*) a resistance to change an initial single pattern (e.g., participant 7) or *ii*) a lack of temporary stability during practice (e.g., participant 4 and 6). For instance, participant 7 showed in the low-speed condition a very strong stability of the coordination pattern 4 with few visits of coordination pattern 9 all along the practice time but without any period of stability of this pattern 9. During practice for this learner, the probability of transiting towards the coordination pattern 4 was very high and kept high during the entire practice period, leading to a linear modeling of those transitions toward coordination pattern 4 even when the DMM modeling of degree 3 allowed for fluctuations. Both degree 1 and degree 3 models, therefore, appeared very similar and the distance between both models was very small, showing a low-level exploration. The low level of exploration of this participant can be explained by his difficulty to leave his initial intrinsic behavior to search for new movement solutions^31^. On the other hand, searching for a new movement solution was potentially not necessary for this participant, as he might have improved superficial parameters like limb speed or acceleration that were sufficient to effectively meet the task requirement^21^.

Focusing on participant 6 on the other side, the low level of exhibited exploration was due to the lack of temporary stability of coordination patterns. This lack of temporary stability was highlighted by the absence of a high probability of emergence of one single pattern at some point during learning (i.e., no pattern with a probability of emergence higher than 0.30. Despite the fact that all the 11 different patterns were visited by this learner, the dynamics of those visits appeared evenly distributed and organized in time (Fig. 3F). Therefore, for each of the 11 patterns, the probabilities of transiting toward a specific coordination pattern remained the same during all the practice, showing a linear modeling of those transitions even if the degree 3 models allowed for fluctuations. Again, both degree 1 and degree 3 models, therefore, appeared to be very similar. In this situation, it appears that it is not difficult to leave an initial intrinsic behavior that limits the level of exploration, but rather the lack of temporary stability that inhibited the learner to effectively explore a new movement solution.

Based on this definition of exploration during learning, the evolution of an initial intrinsic behavior does not occur due to bistability as previously proposed^32^ but rather through metastability (with temporary stability/instability) (Fig. 3E)^33,34^. In a metastable region, "there is attractiveness but, strictly speaking, no attractor"^35^, p172). The presence of a metastable regime during learning may let individuals circumvent the limits of their behavioral dynamics; that is, escape from the initial stability of their intrinsic patterns and explore different coordination patterns with a relative stability^36^. In this way, learners can freely access to different coordination patterns in order to gather information and determine their relevance. Moreover, our results show that during learning, learners can exhibit a new pattern, then return to their original pattern (sometimes for an entire session), then proceed to explore other new patterns. In this context, the relatively stable patterns plays a key role as a "bridgehead"^37^ (p. 391), a refuge coordination patterns from which it is possible to explore and stabilize a new patterns. Exploratory activity during motor learning, therefore, appears to be a cooperation between stability and flexibility^38^. By looking at the probability of transitions between patterns rather than the actual state, the DMM modeling identified this metastability within the learning dynamics, as a subtle blend between period of stability and flexibility between patterns^34^.

Interestingly, due to the high resistance of the water, the level of constraints acting on the learner during underwater locomotion is easy to manipulate. Swimming faster (e.g., from 70% of individual maximal speed to 90% of individual maximal speed), therefore, consists in performing the same task but in a much more constrained environment (i.e., the water resistance being proportional to the speed squared). From the comparison of the level of exploration exhibited between the two speed conditions, no main difference in the quantity of exploration appeared. However, the effect of increased level of constraint in the task appeared only on specific patterns of coordination. More precisely, an increase in speed led to a decrease in the exploration of patterns 8, 9 and 11, accompanied by an increase in the exploration of patterns 2 and 6. Interestingly, by looking at the difference in the level of exploration between each patterns within each speed, it has to be noted that although at low speed only the patterns 1 and 6 showed a different level of exploration (mean difference = 13.87% ± 5.15) as well as the patterns 2 and 10 (mean difference = 14.22% ± 4.41), at high speed twelve differences appeared in the level of exploration between the different patterns, with coordination pattern 6 being more explored than some others and coordination patterns 3, 8, 9 and 11 being less explored than some others.

The level of exploration between the different patterns, therefore, appeared more evenly distributed in low-speed condition than in high-speed condition. Although mechanically in the high-speed condition, it was still possible for the participants to exhibit a coordination between 0 and 360° (i.e., the physical limit was similar in both conditions)^39^, increasing the level of constraint led to a more selective exploration by the learner. Indeed, the global level of exploration was not different between low- and high-speed conditions but some coordination patterns were specifically less explored at high speed compared to low speed to make room for a higher exploration for the other patterns. The effect of the constraint level appears, therefore, more qualitative (i.e., in the selection of patterns that can be explored) than quantitative (i.e., in the global amount of exploration)^40^. When the level of constraint is low, the nature of the patterns explored is wider and individual degeneracy can freely express (i.e., the capacity to exhibit multiple different solutions to achieve a similar task), whereas the increase in the level of constraint really impairs the appearance of some coordination patterns therefore restricting the exploration to fewer coordination patterns^21^. Few patterns then appeared to channel all the exploration around them in highly constrained environments.

From this point, modifying the level and nature of interacting constraint in the task appears to be a useful tool in order to channel the exploration of the learner without decreasing this exploration^41^. Theoretically, if the constraints are managed in the way that the learner’s initial coordination pattern is not a functional motor solution anymore, playing with the constraints can channel the learner to explore new motor solutions (i.e., to destabilize the initial behavior^31^) as well as guide him towards specific new solutions to explore^42^. This later point was supported by the participant 7 (see online appendix B), who showed a very limited amount of exploration in the low-speed condition, both in quantity and in its nature (i.e., a limited number of different patterns explored). Indeed, for this participant, the increase in the level of constraint in the task acted like a perturbation that pushed him out of his initial patterns and allowed him to explore more varied movement solutions.

Interestingly, this restricted nature of the exploratory process seemed to impact the individual rate of learning. Indeed, if we refer to the increase in performance with practice, although a higher frequency in high-speed condition as already been explained in the literature^21,39^, all the participants in the present case showed an increase in performance with practice in both speed conditions. Although a larger improvement of performance was observed in low swimming speed condition, the rate of learning appeared similar between both speed conditions. In fact, considering the inter-individual differences in the rate of learning, it appears that the low-speed condition allowed all the learners to improve their performance at a more similar rate than the high-speed condition.

This result supports the idea that having a large stability region for exploration allows each individual to find its own functional movement solution in the task. This is also supported by the positive correlation between the number of visited patterns and the quantity of exploration, showing that proposing more possibility for exploration to the learner indeed allows more exploration to happen. In fact, even if some patterns are only little explored, it seems beneficial for the learner to still be able to explore them as they may rapidly gather information from their exploration. The initial flexibility of a learner could therefore form the basis for an effective exploration during learning, as recently highlighted by Sidarta et al.^30^. Narrowing the region of exploration by limiting the potential patterns that can be visited during learning can impact the individual dynamics of the performance because the individually necessary information therefore cannot be gathered through an effective exploration^43^. Exploring coordination patterns that do not provide relevant information to the learner to refine the perception-action coupling can, therefore, be less effective in realizing an optimal balance in the use of constraints for a sufficient learning^44^.

A major limitation of this study resides in the absence of a transfer test, as higher exploration during learning is currently expected to impact adaptability or transfer capacity of learners in addition to performance in the training task itself. In other words, a coordination learned through higher exploration may not be more efficient in the present task but more easily transferred to a new but similar task, which could be the major benefit of high exploration during motor learning. Related to this point, the concurrent practice in both high and low speed condition for every learner is also a limitation. Although the practice in different level of constraint was required, the organization of practice in each condition was controlled and regular to avoid impacting the dynamics of learning. Nevertheless, some transfer of learning may have happened between both speed condition which is difficult to avoid but was limited by the different coordination patterns required to perform in each condition. On another point, the rather limited sample size in this experiment limits the possibility to identify optimal search strategies if they exist. While capturing the entire dynamics of learning (i.e., recording every trial/cycle) is time consuming but informative, the highly individual nature of observed learning pathways makes it difficult to find regularities in the search strategies between learners unless potentially a very large sample size can be investigated. Using sensors and automatic processing of the data to capture coordination could help in this regard to increase sample size.

## Conclusion

In this study, we investigated motor learning by identifying three different dynamics of coordination patterns that appeared across practice, through exploration as a metastable regime, i.e., a balance between stability and variability rather than merely transitions between successive patterns. Those exploratory dynamics appeared highly individual both in terms of quantity of exploration and in the nature of the patterns that were explored. Our results showed that a more restrictive set of task constraints applied on the coordination and control solution space did not impair the global quantity of exploration, but rather limited its nature by restraining the different patterns that an individual deeply explore. At the same time, restraining the nature of the different patterns that learners can explore during learning did not impair the learning rate but rather increased the inter-individual differences in this learning rate, showing that more space for exploration can better respect the need for an individual dynamic of exploration. Although modifying constraints during learning can help learners to escape from their initial intrinsic behaviour as well as guide them in their exploration, maintaining enough room for a diversity of coordination patterns to emerge seems key to foster effective exploration of individual movement solutions.

## Methods

### Participants

Initially eight males, all novices in breaststroke swimming, voluntarily participated in this study (mean age = 18.4 years, standard deviation (SD) = 0.7 years). A power calculation for a repeated measures design with 1 group of participants, 4 measurements (2 different conditions at 2 different time of learning), an alpha threshold of 0.05, a target power of 0.8 and a medium to large effect size (η_p_^2^ = 0.19) provided a sample size of 8 participants. A medium to large effect size was expected due to the large number of learning sessions in this protocol^37^. Each participant signed an informed consent form after receiving oral and written descriptions of the procedures, which were approved by the university ethics committee. Participant 3 was not able to compete all the protocols, leaving 7 participants for analysis. The two exclusion criteria were principally related to the validity of subject’s initial breaststroke technique. Importantly, they had to be able to: (a) perform a symmetrical leg kick; and (b) perform leg and arm movements at the same frequency. The swimmers were characterized as being in the first stage of learning (i.e., coordination stage), during which learners still have to establish the basic coordination of the key components of the behaviour^45,46^. All participants had the same goal of learning without any information on *how* to perform.

### General goal of learning and practice sessions

All participants participated in 16 learning sessions. The entire program lasted 8 weeks, with 2 sessions per week. All participants performed at a different time during the day/week, in order to avoid any interaction between participants during the protocol. During each session, in a 25 m indoor pool, participants had to complete 10 × 25 m at sub-maximal speeds (5 trials at 70% of their personal maximal speed (i.e., a comfortable speed, low constraining environment of practice) and 5 trials at 90% of their personal maximal speed (i.e., a high speed, highly constraining environment of practice) (Fig. 7.2). Those sub-maximal speeds were defined during the first session after a familiarization practice of 100 m swim in breaststroke and, thereafter, corresponded to the working speed throughout the entire learning process (i.e., the speed was constant during all the practice sessions). Each session lasted approximatively 35 min per participant and included a 10 min of warm-up (out of water active joints mobilisation and muscular stretching followed by 50 m front crawl swimming) followed by the 10 trials with a start every 2 min 30 s (a trial lasted 30 s followed by a 2 min rest period). Participants were asked to avoid practicing breaststroke during the entire experiment (from the first learning session to the retention test), except during the experimental sessions.

**Figure 7:**
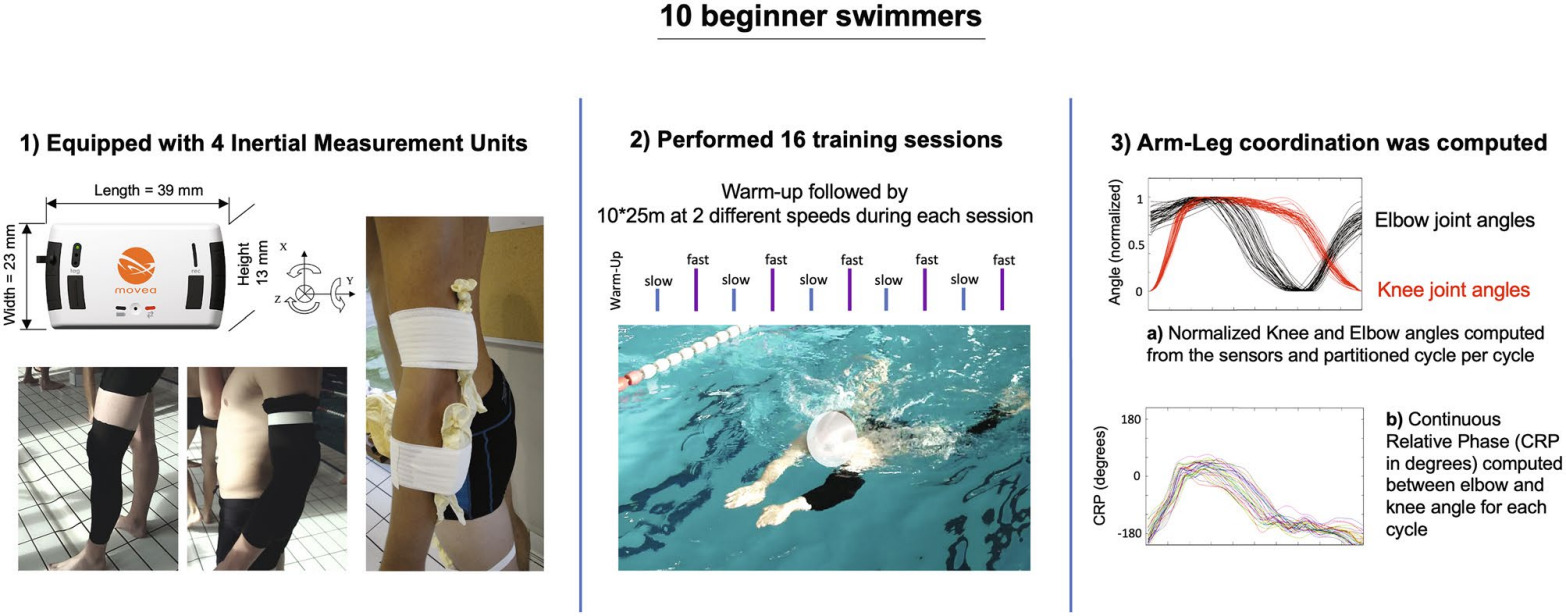
Experimental design. Overview of the experimental design followed by the 10 participants through 3 main steps: 1) material, 2) task, and 3) measurements.

For all the participants, the general goal of learning was to decrease their stroke rate (i.e., number of cycles per second, in Hz) while maintaining the same sub-maximal speed—therefore, increasing their efficiency^47^. Learners were informed of this general goal at the beginning of each session. The basic rules of breaststroke swimming were provided to the participants (as a reminder) during the first session, and only if necessary, thereafter. The speed was self-paced by the learner during the trials based on a target time of competing 25 m. The average speed of performing each trial was measured at the end of each trial and the trial was validated if the actual average speed was within the target speed ± 5% of that speed. If learners failed to follow the rules or the targeted speed, they were stopped by the experimenter and had to perform the trial again. After each trial, learners were informed of their mean stroke rate values (i.e., informed of their performance in the task). No other information was given to the learners during the 16 sessions.

### Data collection

During every sessions, participants were equipped with inertial sensors including 3-D accelerometers, 3-D magnetometers and 3-D gyroscopes (MotionPod3, Movea, Grenoble, France)^48^ (Fig. 7.1). The acquisition frequency of the sensors was 100 Hz. Four motion sensors were positioned on the left side of the swimmers, respectively on the forearm (posterior surface of the proximal portion), the arm (posterior surface of the distal portion), the thigh (anterior surface of the distal portion), and the leg (anterior surface of the proximal portion), in order to have the sensors in direct contact with a bony part of the limb. At the beginning of each session, the position of the motion sensors was placed on a black marker, which defined the location of the sensor from the last session. Swimsuit was also worn on the two limbs where sensors were placed in order to limit resistances due to the presence of the sensors. Once the swimmer was ‘suited-up’, he entered the second lane in the pool (i.e., at least 2 m far from the wall to avoid any magnetic disturbance) and performed the 10 trials. Once the trials were completed, the data were uploaded and synchronized a posteriori with Matlab r2015a (version 8.5.0, The MathWorks Inc., Natick, MA, USA).

### Processing of behavioural data

Thereafter, elbow and knee angles were computed for each trial by calculating the relative angle between two sensors. Time series representing knee and elbow angles were then computed. These time series were filtered with a low-pass Fourier filter (cut-off frequency 8 Hz^24^) and partitioned cycle per cycle (i.e., one cycle beginning with a maximal knee flexion and finishing with the next maximal knee flexion). The first cycle as well as the last cycle were removed to account for acceleration of swimming speed due to push-off the wall or deceleration when approaching the wall. For each trial, knee and elbow angular positions for 3 to 17 cycles were normalized between 0 and 1 and used for characterizing the inter-limb coordination of the swimmer (Fig. 7.3).

The nature of the behaviour was derived from the arm-leg coordination and was assessed by the Continuous Relative Phase (CRP) between knee and elbow angles. The CRP was computed based on elbow and knee angles in the same way as previous experiments^24,39^, which has been shown to be an effective parameter to quantify the nature of swimmer’s behaviour. A typical in-phase behaviour (i.e., a relative phase value close to 0°) represents an inefficient swimming technique consisting in simultaneous arm propulsion and leg recovery, and conversely an effective coordination pattern has been characterized by a more complex coordination within a single cycle, with fluctuations of relative phase between anti-phase (i.e. a value of − 180°) to in-phase coordination mode to anti-phase (see^24^ for a precise description of an effective coordination pattern).

### Processing of performance data

During each trial, the instantaneous stroke frequency (Hz) was recorded for each cycle from the cycle duration (measured with the motion sensors) following the equation f = 1 / cycle duration (s). Therefore, changes in performance were defined by the decrease in stroke frequency cycle after cycle. The average frequency value per session was computed for each individual and modelled with an exponential function^49^. The exponential function used to fit the movement performance over sessions is shown in Eq. 1:

1 $${\text{f}}\left( t \right) \, = {\mathbf{a}}*{\text{ exp}}( - {\mathbf{b}}*t) \, + {\mathbf{c}}$$

where *t* is the practice time (session number), **c** represents the asymptotic performance, **a** represents the initial performance (when *t* = 0, exp(-b*t) = 1 and **a** + **c** represents the performance level before practice), finally **b** represents the learning rate. From this model, the higher is the value of **b**, the faster is the learning rate (i.e., representing an early rapid increase in performance followed by a later slow increase). The quality of the fitting is presented by r^2^ and Root Mean Square Error (RMSE)^50^.

### Profiling motor coordination

An unsupervised cluster analysis procedure was used in order to differentiate the patterns of coordination exhibited by the learners^19^. The time series of CRP from the cycles of all the seven participants in both speed conditions were used to compute the cluster analysis (i.e., all the cycles, all the sessions, all the participants). Such a cluster analysis allows partitioning the entire set of cycles into meaningful sub-groups or clusters, whereas the “real” number of groups is unknown a priori. The Fisher-EM algorithm has been used for the present experiment^51,52^. The Fisher-EM algorithm is an iterative cluster algorithm that projects the data in a new subspace at each iteration in such way that emerging clusters maximize the Fisher information (i.e., maximize the inter-cluster distance while minimizing the intra-cluster distance). The final number of emerging clusters (n) was selected based on the Bayesian Information Criterion (BIC) for model selection^53^ with the first value of the plateau representing the model that best represents the data (also known as the “elbow” method). The ordering of the clusters (from 1 to n clusters) is done randomly by the algorithm based on a random initiation.

### Quantifying motor exploration

From the clustering, each trial was labelled with a specific exhibited coordination profile. The time series of those exhibited coordination were re-constructed putting one cycle after the previous one in the chronological order they were performed, representing the successive behaviours that were exhibited by a learner^18,30^. Those time series of labelled behaviours were thereafter modelled using Drifting Markov Models (DMM)^54^. DMM have been used in the genome literature and are a relevant tool for modelling how qualitative patterns are organized in time. Specifically, modelling DNA sequences with stochastic models and developing statistical methods to analyse the DNA sequencing have been challenging questions for statisticians, and the most popular model in this domain is the Markov model on the nucleotides (i.e., the modelling of *a c g t* nucleotides).

Although a traditional Markov model gives a broad overview of the main transitions occurring between the different patterns within the whole time-series of patterns (i.e. a transition matrix), Vergne^54^ developed a DMM that varies the transition matrix between the different patterns on the basis of a predetermined polynomial model with a degree *n* that is set by the experimenter. In other words, this modelling is meant to provide the evolution in time of the original transition matrix (e.g., the change in time in the probability of the transitions converging towards a specific pattern). Eventually, using the DMM to model the evolution of the transitions between patterns provides a modelling that accounts for the probability of appearance of any potential patterns as learning operates. DMM are applied individually on the time series of clusters of each participant (i.e., only on the clusters individually exhibited by each participant), for modelling the probability of appearance of one pattern of coordination regarding the previous pattern.

The order of the Markov chain was set to one (i.e., only one previous cycle was considered for the transition), however, two models with different degrees were applied for the modelling of the evolution of the transitions between patterns of coordination. On one hand, a model of degree one was applied, representing a linear trend of the transition matrix. Indeed, a linear trend represents linear increase or decrease of the probability of transition from one coordination toward the final to-be-learned coordination. In other words, with such a linear model, each time a learner practices, he increases the probability of appearance of the to-be-learned coordination.

A model of degree three was applied on the time series. This model, based on the use of the BIC and Akaike Information Criteria (AIC) for model selection^53^, represented the model that best fitted the actual data and was considered as representing the actual fluctuations that occurred during learning (see Online Appendix Afor the BIC and AIC values for all possible models). Eventually, the average Euclidean distance between those two models (i.e., degree 1 and degree 3) was computed for each coordination pattern for each learner. This distance between the degree one model (i.e., linear modelling of the probabilities of transition) and the degree three model (i.e., the actual evolution of the probabilities) therefore quantify the nature of the exploration. A large distance between the linear and the degree three model reflects the presence of high exploration of a specific pattern at some point during learning (or specific patterns successively), in other words strong *anchor points* in the learning dynamics. A small distance between both models represents an absence of anchor points in the learning dynamics and a more linear appearance/disappearance of the coordination patterns (i.e., the probability of appearing/disappearing of the pattern evolving linearly).

### Summary of dependent variables

With reference to the performance indicator, both the decrease in stroke frequency between the first session and the last session as well as the rate of learning (i.e., value of **b** from the exponential models) were considered. In regard of the analysis of motor exploration, after the clustering and the DMM application, the quantity of exploration in percentage (i.e., the distance between two models) for each cluster is considered per participant and speed condition. The standard deviation (SD) of the quantity of exploration both within participants and within speed conditions is presented in order to reflect the within individual and within speed amount of variability of the exploratory processes.

### Statistical analysis

With reference to the analysis of the increase of performance between the first session and the last session, after normality and homogeneity of variance were checked, a two-way ANOVA (within-subject effects: session time [first; last] * speed condition [low; high]) was performed on the stroke frequency values between the time of testing and the two speeds of swim. Concerning the rate of learning (i.e., the value of **b** from the exponential models) and the variability in exploration, a paired sample *t*-test was performed to compare the rate of learning and the SD of the quantity of exploration between the two speed conditions. When the difference was significant, Cohen’s *d* was computed as a measure of the size of the effect, with *d* = 0.2 representing a small effect, *d* = 0.5 representing a medium effect and *d* = 0.8 representing a large effect^55^.

With regard to the motor exploration quantity, a two-way ANOVA was performed (within-subject effects: cluster [1–11] * speed condition [low; high]) in order to investigate any difference in the quantity of exploration of a specific cluster and any difference due to the speed condition. When necessary, the *p* values were corrected for possible deviation from sphericity using the Greenhouse-Geisser correction when the mean epsilon was lower than 0.75. Otherwise, the Hyun-Feld procedure was used. When a significant effect appeared, post-hoc test using Bonferroni correction were used. Partial eta squared (η_p_^2^) was calculated as an indicator of effect size, considering that η_p_^2^ = 0.02 represents a small effect, η_p_^2^ = 0.13 represents a medium effect and η_p_^2^ = 0.26 represents a large effect^55^. As one participant dropped out during the experiment, achieved power was also calculated and informed when necessary for the tests.

Concerning the relationship between the emerging number of patterns, average individual quantity of exploration and performance improvement and rate, Pearson correlations were used when the assumption of normality was met, otherwise Spearman’s rho correlation was computed. All tests were performed using JASP Statistics V0.13.1 (July 2020—www.jasp-stats.org), with a level of statistical significance fixed at *p* =  < 0.05.

## Supplementary Information

Supplementary Information.

## Data Availability

Data supporting the results reported in the article can be found in National Institute of Education data repository: https://researchdata.nie.edu.sg/dataset.xhtml?persistentId=doi:10.25340/R4/1UBOTA.
