# Supplementary material for: Narrowing the coordination solution space during motor learning standardizes individual patterns of search strategy but diversifies learning rates
Source: Sci Rep. 2023 Feb 3;13:2009. doi: 10.1038/s41598-023-29238-z (PMC9898268; doi:10.1038/s41598-023-29238-z)
Supplement: Supplementary file 1 — Supplementary Information. [file 41598_2023_29238_MOESM1_ESM.docx]

**Appendix A:** BIC and AIC for the different potential DMM models

**Appendix B:** individual exploration (A) and modelling with degree 1 model (B) and degree 3 model (C) in both speed conditions and for each participant
